# Supplementary material for: End Sequence Analysis Toolkit (ESAT) expands the extractable information from single-cell RNA-seq data
Source: Genome Res. 2016 Oct;26(10):1397–410. doi: 10.1101/gr.207902.116 (PMC5052061; doi:10.1101/gr.207902.116)
Supplement: Supplemental Material [file supp_gr.207902.116_Supp_Materials.tar.gz › ESAT-esat_v0.1/doc/html/classbroad_1_1core_1_1sequence_1_1_fasta_quality_binary_i_o-members.html]

ESAT: Member List


ESAT


- Main Page
- Related Pages
- Packages
- Classes
- Files

- Class List
- Class Index
- Class Hierarchy
- Class Members

All Classes Namespaces Files Functions Variables Enumerator Pages

- broad
- core
- sequence
- FastaQualityBinaryIO

broad.core.sequence.FastaQualityBinaryIO Member List

This is the complete list of members for broad.core.sequence.FastaQualityBinaryIO, including all inherited members.

|  |  |  |
| --- | --- | --- |
| BUFFER\_SIZE | broad.core.sequence.FastaQualityBinaryIO | static |
| createAsciiFromBinary(String binaryQualityFile, int numQualBasesPerRow) | broad.core.sequence.FastaQualityBinaryIO |  |
| createBinaryFromAscii(InputStream in, String outFile) | broad.core.sequence.FastaQualityBinaryIO |  |
| getQualityBases(Connection conn, String table, String seqId, int start, int numOfBases) | broad.core.sequence.FastaQualityBinaryIO |  |
| getQualityBases(Connection conn, String table, String seqId) | broad.core.sequence.FastaQualityBinaryIO |  |
| SEQ\_ID\_REC\_LENGTH | broad.core.sequence.FastaQualityBinaryIO | static |
| toDBFromAscii(InputStream in, final Connection conn, String table) | broad.core.sequence.FastaQualityBinaryIO |  |


---

Generated on Fri Aug 15 2014 15:10:55 for ESAT by  

 1.8.7
